# Alzheimer’s Disease Risk Reduction: An Evidence-Based, Multi-Component Lifestyle Program, Pre- & Intra-Covid-19

**DOI:** 10.1093/geroni/igab046.2435

**Published:** 2021-12-17

**Authors:** Gina Touch, Paul Bendheim

**Affiliations:** University of Arizona College of Medicine Phoenix, Phoenix, Arizona, United States

## Abstract

Alzheimer’s disease (AD) is the 5th leading cause of death in the USA. With nearly 300 failed therapeutic trials to date, lifestyle modifications have been shown to reduce AD risk by as much as 50%. Preceding the FINGER Study by nearly a decade, the BrainSavers Brain+Body Total Fitness program was developed by an interdisciplinary team to reduce the risk of AD / all-cause dementia and promote healthy aging via education, exercise, and engagement. This evidence-based program utilizes the principles of neuroplasticity and cognitive reserve. Pre-Covid, BrainSavers was delivered live, led by certified instructors. Two years of curriculum were developed, comprised of six lifestyle components: cognitive exercise, physical exercise, healthful nutrition, socialization, stress reduction, and sleep hygiene. Results of a six month beta trial documented self-rated improvements in memory and general cognitive performance, quality of life, socialization, nutritional status, and physical fitness. Quantitative results showed statistically significant differences in physical fitness measures including cardio-respiratory endurance, lower body strength, balance, speed, and agility. Trends were seen in six of nine cognitive skills. During Covid the program was transformed into an online format as BrainSavers Synapse: Staying Connected, which has been enthusiastically received. Future research will compare longer-term outcomes of both formats. Based on results to date and extensive peer-reviewed literature on lifestyle as a modifier of dementia risk, we predict this program will contribute to better individual and societal outcomes, including substantial improvements in cognitive and overall health, and a significant reduction in healthcare costs.

